# An Efficient In Vitro Propagation Protocol of Cocoyam [*Xanthosoma sagittifolium* (L) Schott]

**DOI:** 10.1100/2012/346595

**Published:** 2012-04-30

**Authors:** Anne E. Sama, Harrison G. Hughes, Mohamed S. Abbas, Mohamed A. Shahba

**Affiliations:** ^1^Department of Horticulture & Landscape Architecture, Fort Collins, CO 80523-1173, USA; ^2^Department of Natural Resources, Institute of African Research and Studies, Cairo University, Giza 12613, Egypt

## Abstract

Sprouted corm sections of “South Dade” white cocoyam were potted and maintained in a greenhouse for 8 weeks. Shoot tips of 3–5 mm comprising the apical meristem with 4–6 leaf primordial, and approximately 0.5 mm of corm tissue at the base. These explants were treated to be used into the culture medium. A modified Gamborg's B5 mineral salts supplemented with 0.05 **μ**M 1-naphthaleneacetic acid (NAA) were used throughout the study. Thidiazuron (TDZ) solution containing 0.01% dimethyl sulfoxide (DMSO) was used. Erlenmeyer flasks and test tubes were used for growing cultures. The effect of different media substrate, thidiazuron, and the interaction between TDZ and Benzylaminopurine (BAP) on cocoyam culture were tested. Results indicated that cocoyam can be successfully micropropagated in vitro through various procedures. All concentrations tested (5–20 **μ**M BAP and 1–4 **μ**M TDZ) produced more axillary shoots per shoot tip than the control without cytokinins. Greater proliferation rates were obtained through the use of 20 **μ**M BAP and 2 **μ**M TDZ, respectively, 12 weeks from initiation. Shoots produced with BAP were larger and more normal in appearance than those produced with TDZ, which were small, compressed, and stunted. The use of stationary liquid media is recommended for economic reasons.

## 1. Introduction

Cocoyam [*Xanthosoma sagittifolium *(L) Schott] is an herbaceous, monocotyledonous crop that belongs to *Araceae* family. The stem is a starch-rich underground structure, the corm, from which offshoots called cormels develop. Flowering is rare, but when it occurs, the inflorescence consists of a cylindrical spadix of flowers enclosed in a 12–15 cm spathe [1]. It is a staple food in the tropics and subtropics and one of the six most important root and tuber crops worldwide [2]. The corm, cormels, and leaves of cocoyam are an important source of carbohydrates for human nutrition, animal feed [3–5], and of cash income for farmers [6]. Africa produces about 75% of the world production which is about 0.45 million tons [7]. Cocoyam production requires high labor and water. Also, its breeding is difficult in addition to its sensitivity to diseases and pests [8].

Cocoyam usually propagates vegetatively from tuber fragments, which increase pathogens distribution. Vegetatively propagated commercial varieties are highly susceptible to the cocoyam root rot disease caused by *Pythium myriotylum *[9], and Dasheen mosaic virus that is found in the leaves, corm, and cormels [10]. Trials have been made using conventional procedures to rapidly increase cocoyam-planting material. Micropropagation is an efficient method to mass propagate good-quality materials that substantially improves production. It involves the use of defined growth media supplemented with appropriate growth regulators that enable morphogenesis to occur from naturally growing plant parts. This helps in producing a large number of plants from a single individual in short time and in limited space [11]. Previous studies have shown that shoot multiplication, somatic embryogenesis, and tuberization could be induced in shoot tips of cocoyam cultured in vitro on Murashige and Skoog medium [12] supplemented with various combinations of indol butyric acid (IBA), 1-naphthalene acetic acid (NAA), 2,4-dichlorophenoxyacetic acid (2,4-D), Benzylaminopurine (BAP), and kinetin [13].

The biochemical aspects of induction of in vitro organogenesis have been investigated in a number of plants including carrot [14], pea [15], summer squash [16, 17], winter squash [18], soybean [19], taro [20], watermelon [21], groundnut [22], asparagus [23], black pepper [24], canola [25], cotton [26], date palm [27], lentil [28], common bean [29], sunflower [30], rice [31], and banana [32]. In spite of its importance in many countries, cocoyam has received very little research attention and is considered insufficiently studied crop [33]. According to Goenaga and Chardon [34], the yield potential of cocoyam is seldom realized, mainly because of a lack of knowledge concerning diseases, proper management practices, and physiological determinants that may limit plant growth and development. In this respect, this study will hopefully contribute to a sustainable cocoyam production. Although it was proposed that large numbers of cocoyam could be produced in vitro, the techniques were not adequately standardized for routine micropropagation. Therefore, the objective of this work is to verify and improve micropropagation of cocoyam via axillary shoot proliferation.

## 2. Materials and Methods

### 2.1. Source of Explants

Cocoyam “South Dade” white plants were obtained from the Tropical Fruit Company, Homestead, FL as sprouted corm sections. Each of these sections was potted in polyethylene pots (*≅*100 cm^2^) in a mix of peat, perlite, and vermiculite (1 : 1 : 0.5 by volume). These plants were maintained in a greenhouse under natural photoperiods. Temperature was maintained at 23 ± 2°C. Plants were watered as needed with tap water and fertilized with liquid fertilizer containing N : P : K at 20 : 10 : 20 by volume twice a week. After 8 weeks of planting, sprouts were collected, trimmed to about 5 cm, and washed under running tap water for 30–60 minutes. These were further excised to finally obtain shoot tips of 3–5 mm comprising the apical meristem with 4–6 leaf primordial, and approximately 0.5 mm of corm tissue at the base. These explants were disinfected in a laminar flow hood before transferred into the culture medium.

### 2.2. Basal Medium (BM)

A modified Gamborg's B5 mineral salts [35] supplemented with 0.05 *μ*M 1-naphthaleneacetic acid (NAA) were used throughout the study. The modified component of B5 microsalts was MnSO_4_·4H_2_O at 10 mg L^−1^. Organics consisted of myo-inositol (100 mg L^−1^), thiamine HCl (10 mg L^−1^), nicotinic acid (1 mg L^−1^), and pyridoxine HCl (10 mg L^−1^). Sucrose was provided at 30 g L^−1^ as a source of carbon and energy. Whenever a semisolid medium was desirable, agar (Sigma agar, type A) was added at a concentration of 0.4%. The pH of the medium was adjusted to 5.7 ± 0.02. Thidiazuron (TDZ) solution containing 0.01% dimethyl sulfoxide (DMSO) was used. Erlenmeyer flasks (125 mL) and test tubes (25 × 150 mm) were used for growing cultures. Aliquots of 25 mL and 15 mL were dispensed into the flasks and test tubes, respectively. Flasks were stoppered with nonabsorbent cotton plugs, and then covered with aluminium foil. Test tubes were covered with polypropylene closures, Kaput caps (Bellco Glass, Inc., NJ, USA). The media-containing vessels were then autoclaved for 18 minutes at 121°C.

### 2.3. Explant Establishment and Multiplication

To test the effect of different media substrate, solid or liquid with the most efficient shaking pattern, explants were initiated on three media supplemented with either 5.0 *μ*M Benzylaminopurine (BAP), 20.0 *μ*M BAP, or 2.0 *μ*M TDZ. Each medium was either solidified with 0.4% agar or maintained in the liquid state. Liquid media were either continuously shaken on a rotary shaker (Model New Brunswick Scientific, Edison, N. J.) at 80 rpm, held stationary but with the suspension of the explants in the medium, or held stationary with the tissue supported on a filter paper (Whatman no. 1) bridge. Treatments were replicated 10 times, and the whole experiment was repeated twice. Cultures were monitored biweekly and rated from 1 to 4 for survival frequency and shoot elongation, where 1 = creamy or dead cultures with no apparent growth, 2 = growth initiation and appearance of green coloration, 3 = increase in growth, green coloration, and leaf differentiation, and 4 = development of healthy green leaves.

To test TDZ effect on multiplication, the BM was supplemented with TDZ at levels of 1.0, 2.0, 4.0, and 8.0 *μ*M as well as with 5 *μ*M BAP which served as the control. Test tubes of stationary liquid media, without any form of support, were used in all cases. The treatments were replicated 10 times. Explants of 3–5 or 6–10 mm were used. Shoot length, base diameter, and number of axillary shoots as well as roots formed per culture were monitored biweekly for six weeks.

To test the effect of the interaction between TDZ and BAP on cocoyam culture, the explants were cultured on agitated liquid media using six treatments of BAP at 0.0, 10.0, and 20.0 *μ*M factorially combined with TDZ levels of 0.0 and 2.0 *μ*M and supplemented with 0.05 *μ*M NAA. The cultures were replicated 20 times and were maintained in their various initiation media for six weeks. They were monitored biweekly for shoot length, base diameter, axillary shoots, and adventitious root formation. At the end of the initiation phase, shoots were trimmed of any axillary shoots to ensure uniformity and transferred into two media for proliferation. These multiplication media consisted of BM supplemented with either 20.0 *μ*M BAP or 2.0 *μ*M TDZ. Cultures were monitored for eight weeks, principally for the formation of axillary shoots and adventitious roots in addition to shoot length and base diameter. A second subculture was made into fresh media with microshoots serving as explants. Culture was either maintained in their respective treatments on shakers, or subcultured into semisolid media in test tubes. The latter cultures were derived from the 2.0 *μ*M TDZ treatment only. Cultures in the semisolid media were treated in two different ways. They were either maintained in the same media or were subcultured into one that was hormone-free. After six weeks, microshoots from the semisolid media were subsequently subcultured into BM and hormone-free media contained in flasks and test tubes. Cultures were incubated for four weeks, and data were collected on shoot and root formation at two-week intervals. Using another method, cultures were initiated on stationary liquid media in test tubes, and the source material was six week old tissue culture-regenerated plants grown in the greenhouse. The test media consisted of BM supplemented with BAP at 5.0 and 10.0 *μ*M in factorial combinations with 0.0, 1.0, 2.0, and 4.0 *μ*M TDZ. Also, cultures were initiated on stationary liquid media in Erlenmeyer flasks. This medium consisted of BM supplemented with 5.0 *μ*M BAP. BAP levels used were 0.0, 5.0, 10.0, and 20.0 *μ*M. These levels were combined with TDZ at 0.0, and 2.0 *μ*M in factorial design.

All cultures were incubated in growth chambers maintained at 25 ± 3°C under continuous illumination. These culture conditions were the same for each of the different stages, with slight differences associated with location within the growth room. Light intensities were measured with an LI-185 Quantum/Radiometer/Photometer (Lambda Instruments Corp., Lincoln, Nebraska).

### 2.4. Data Analysis

Experiments were laid out as a complete block design. All data were subjected to an analysis of variance using unequal replications where contamination was observed. Treatment means were separated by Tukey's Multiple Range Test at a 5% level of significance (SAS Institute, 2006).

## 3. Results

### 3.1. Media Substrate Effect on Cocoyam Culture

**Figure 1:**
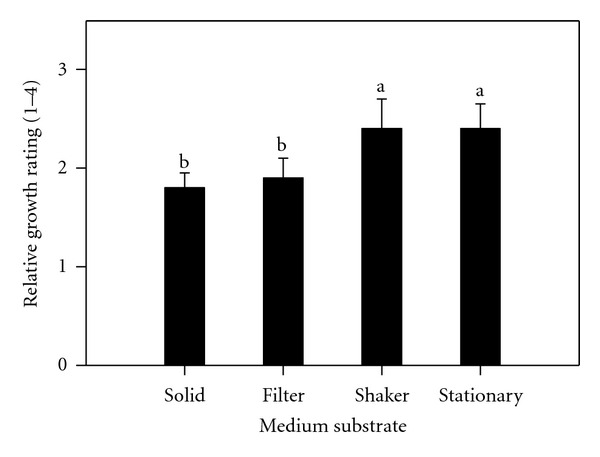
Effect of substrates (solid, liquid filter, liquid shaker and liquid stationary) on relative growth of cocoyam shoot tips cultured on growth regulator treatments after 4 weeks of initiation. Relative growth was rated on a scale of 1–4, with 4 be the highest growth. Columns labeled with the same letter are not significantly different at *P* = 0.05 using Multiple Range Test. Vertical bars at the top represent standard errors.

**Figure 2:**
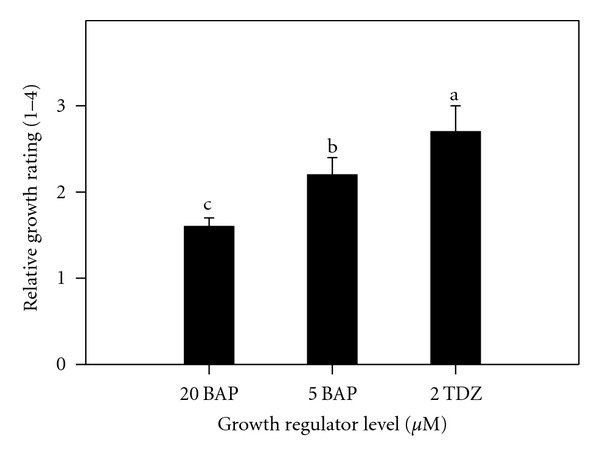
Effect of growth regulator levels on relative growth of cocoyam shoot tips cultured on medium substrates after 4 weeks of initiation. Relative growth was rated on a scale of 1–4, with 4 be the highest growth. Columns labeled with the same letter are not significantly different at *P* = 0.05 using Multiple Range Test. Vertical bars at the top represent standard errors.

Generally, the disinfection procedures resulted in low contamination ranging from none in solid media to only 7.0% in agitated and stationary media. All explants survived as evidenced from their enlargement and manifested by an elongation of the shoot tip and a swelling of the base by the second week of culture. At the same time, most cultures changed from the initial creamy color to green coloration. Analysis of variance indicated a significant difference among different medium substrates and among different growth regulator treatments during the initiation of cocoyam tissue cultures (Table 1). Initiation on liquid culture, either on shaker or held stationary, was better than filter bridges or solid medium (Figure 1). Evaluations indicated that 2.0 *μ*M TDZ and 5.0 *μ*M BAP are important in the initiation of cocoyam tissue cultures. Comparisons indicated that 2.0 *μ*M TDZ was significantly better with 5.0 *μ*M BAP intermediate than 20.0 *μ*M BAP which inhibited shoot elongation (Figure 2).

### 3.2. Media Substrate Effect on Cocoyam Multiplication

**Figure 3:**
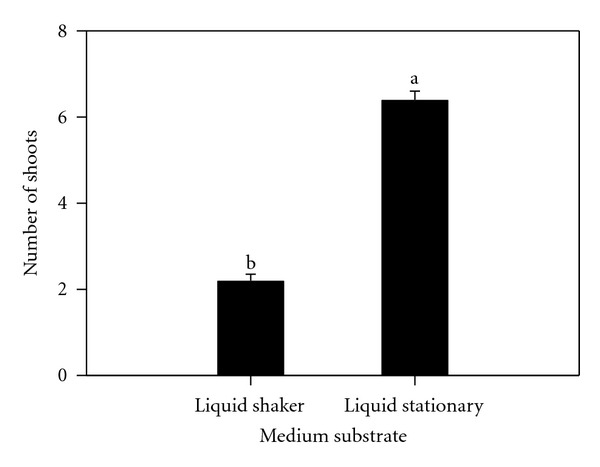
Effect of substrates (liquid shaker and liquid stationary) on number of shoots multiplied on different growth regulator levels after 6 weeks of subculture. Columns labeled with the same letter are not significantly different at *P* = 0.05 using Multiple Range Test. Vertical bars at the top represent standard errors.

**Figure 4:**
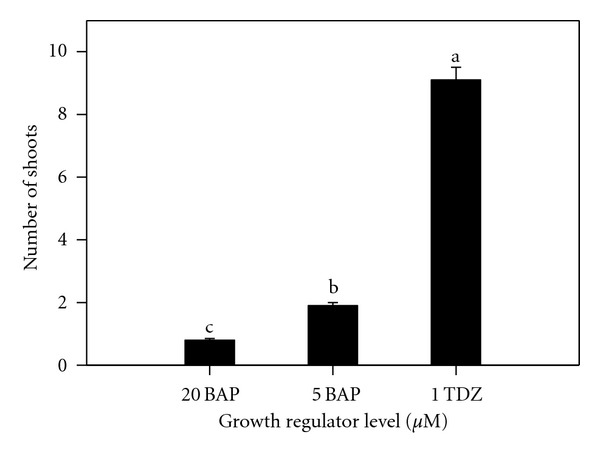
Effect of growth regulator levels on the numbers of cocoyam shoots multiplied on liquid substrates after 6 weeks of subculture. Columns labeled with the same letter are not significantly different at *P* = 0.05 using Multiple Range Test. Vertical bars at the top represent standard errors.

Analysis of variance indicated a significant difference in the number of axillary shoots per shoot tip between stationary liquid and agitated liquid media (Figure 3), among the growth regulators treatments (Figure 4) and their interaction. The highest average number of shoots per shoot tip (9.1) was obtained with 1.0 *μ*M TDZ treatment as compared to 5.0 *μ*M BAP (1.9) and 20.0 *μ*M BAP (0.8).

### 3.3. Thidiazuron Influence on Shoot-Tip Initiation

Analysis of variance indicated no significant difference in number of cocoyam shoots by the second, fourth, or even sixth week of culture, among different TDZ concentrations (1.0, 2.0, 4.0, and 8.0 *μ*M) as compared with 5.0 *μ*M BAP as a control treatment. Initial size of the explants affected its growth and development. The larger explants (6–10 mm) established and developed faster that smaller ones (3–5 mm).

### 3.4. Interaction Effect of TDZ and BAP on Cocoyam Culture on Agitated Liquid Media

Results indicated that the rate of shoot proliferation was slow under all six treatments during the six weeks of culture initiation with no significant difference among treatments. In multiplication media, the proliferation rate was low under all treatments for the first two weeks, but increased by the fourth and sixth weeks (Figure 6). Analysis of variance indicated that 20.0 *μ*M BAP had a better effect on roots number and shoot length while 2.0 *μ*M TDZ had a better effect on shoots number after 6 weeks. The effect of both TDZ and BAP was similar after 4 weeks of culture. After six weeks of culture, 20.0 *μ*M BAP achieved an average shoots number of 9.7, an average roots number of 6.0, and an average shoot length of 75.0 mm while 2.0 *μ*M TDZ achieved an average shoots number of 13.1, an average roots number of 1.7, and an average shoot length of 65.1 mm.

**Figure 5:**
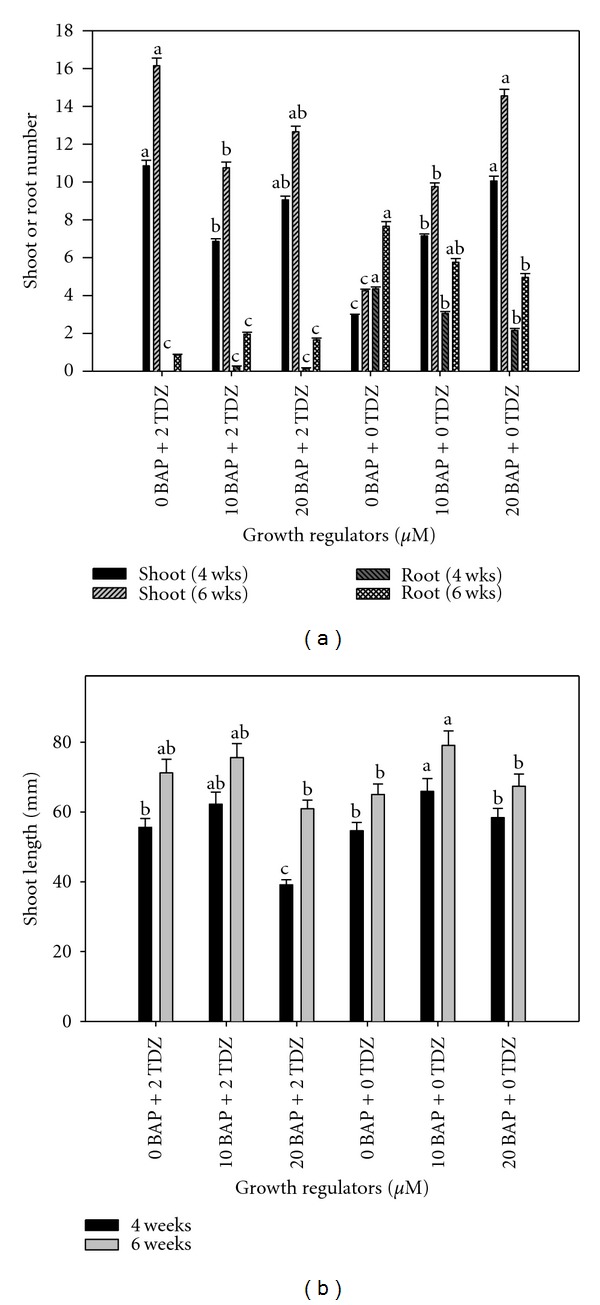
Effect of initiation growth regulator treatments on cocoyam shoot proliferation, root formation (a) and shoot length (b) during multiplication. Columns labeled with the same letter are not significantly different at *P* = 0.05 using Multiple Range Test for treatment effect comparison at 4 and 6 weeks. Vertical bars at the top represent standard errors.

Analysis of variance indicated a significant effect of different growth regulators treatments in the initiation culture during the multiplication phase (Table 2). The average number of shoots per culture ranged from 3.0 in the cytokinin-free control to 10.9 in culture with 20.0 *μ*M BAP as well as the culture with 20.0 *μ*M BAP plus 2.0 *μ*M TDZ after 4 weeks of culture (Figure 5(a)). After 6 weeks, it ranged from 4.3 in the control to 16.2 in culture with 2.0 *μ*M TDZ. Roots number and shoots length were similarly affected by the growth regulators treatments. The control treatment had the best effect on roots number (7.7) followed by 10.0 *μ*M BAP (5.8) and 20.0 *μ*M BAP (5.0) (Figure 5(a)). Also, 10.0 *μ*M BAP achieved the highest average shoot length (79.3 mm) (Figure 5(b)).

**Figure 6:**
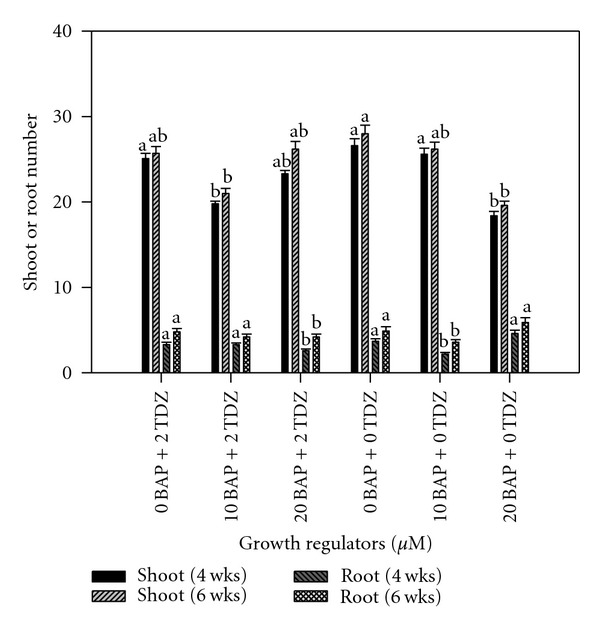
Effect of initiation growth regulator treatments on cocoyam shoot and root proliferation, after subculturing twice. Columns labeled with the same letter are not significantly different at *P* = 0.05 using Multiple Range Test for treatment effect comparison at 4 and 6 weeks. Vertical bars at the top represent standard errors.

After subculturing twice, shoot proliferation and root formation were negatively correlated (*r* = −0.65 and −0.57 after 4 and 6 weeks resp.). Mean number of axillary shoots ranged from 18.5 with 20.0 *μ*M BAP to 26.7 with the cytokinin-free control after 4 weeks and from 19.7 with 20.0 *μ*M BAP to 28.7 with the control treatment after six weeks (Figure 6). The superiority of BAP to TDZ on root formation was evident. Shoots proliferated on BAP developed roots after the second week in culture. The poor rooting ability in TDZ as compared to the previous multiplication phase indicated its repressive effect on rooting. Root number ranged from 2.3 and 3.7 for 10.0 *μ*M BAP to 4.7 and 6.0 for 20.0 *μ*M (Figure 6).

### 3.5. Interaction Effect of TDZ and BAP on Cocoyam Culture on Stationary Liquid Media in Test Tubes

After six weeks of initiation on stationary liquid media, a few shoots developed and there was no significant treatment effect. The average number of new shoots ranged from 0.1 for the joint effect of 10.0 *μ*M BAP and 1.0 *μ*M TDZ to 0.7 for the cytokinin-free control. The highest average root number was produced with the control (9.6), with 100% rooting followed by 53% with 5.0 *μ*M BAP and 6.7% with 10.0 *μ*M BAP.

**Figure 7:**
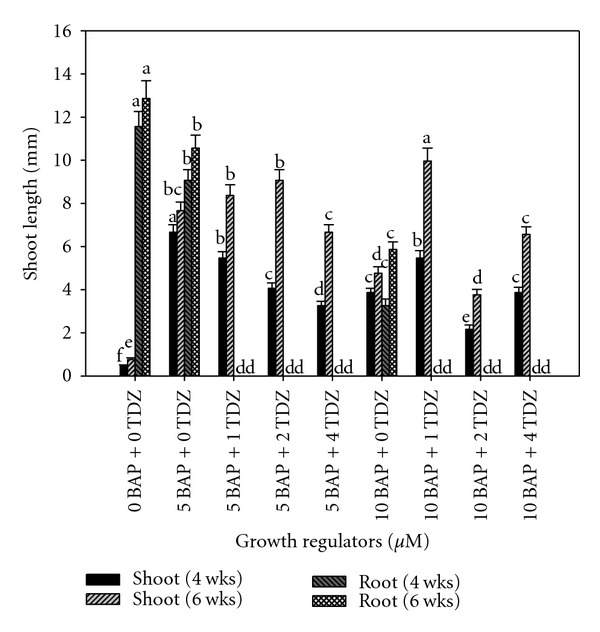
Interaction effect of TDZ and BAP on cocoyam axillary shoot multiplication and root formation on stationary liquid media in test tubes. Columns labeled with the same letter are not significantly different at *P* = 0.05 using Multiple Range Test for treatment effect comparison at 4 and 6 weeks. Vertical bars at the top represent standard errors.

Analysis of variance indicated a significant difference among treatments after 4 weeks in the multiplication media. The average number of shoots per culture was 0.5 in the cytokinin-free control and was 3.9 in a media with 10.0 *μ*M BAP while it was 6.7 with 5.0 *μ*M BAP (Figure 7). At six weeks, the pattern of shoot production changed when the greatest number of shoots was produced in 10.0 *μ*M BAP combined with 1.0 *μ*M TDZ. Generally, there was an approximate doubling of shoot numbers from four to six weeks in all combinations of TDZ with BAP (Figure 7). Growth regulators levels significantly affected rooting, and there was no significant difference in roots numbers between week four and week six. TDZ completely suppressed root formation (Figure 7).

### 3.6. Interaction Effect of TDZ and BAP on Cocoyam Culture on Stationary Liquid Media in Erlenmeyer Flasks

**Figure 8:**
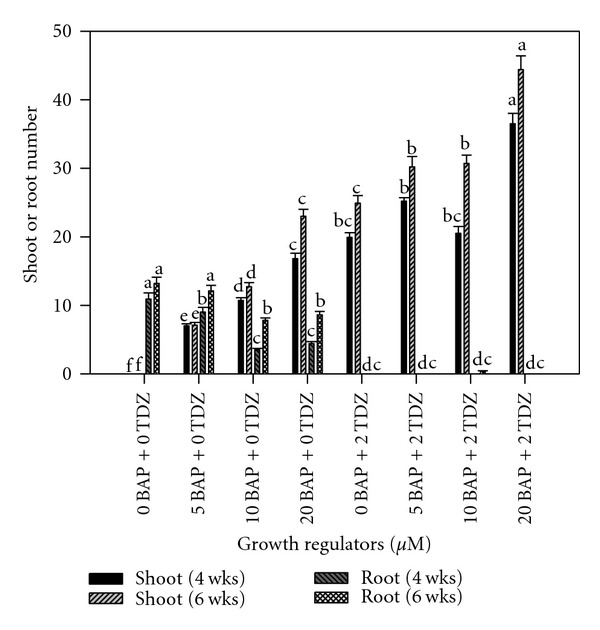
Interaction effect of TDZ and BAP on cocoyam shoot and root proliferation after reculturing in stationary liquid media in Erlenmeyer flasks. Columns labeled with the same letter are not significantly different at *P* = 0.05 using Multiple Range Test for treatment effect comparison at 4 and 6 weeks. Vertical bars at the top represent standard errors.

Explants subcultured on Stationary Liquid Media in Erlenmeyer Flasks proliferated heavily. After 4 weeks of subculture, an average of 36.6 shoots per culture were formed in media containing 20.0 *μ*M BAP combined with 2.0 *μ*M TDZ. Shoot proliferation increased two weeks later in the same order, where an average of 44.5 shoots was produced with same previously mentioned combination (Figure 8).

An average of 11 roots was formed per culture after 4 weeks of subculture, and 13.3 roots were formed after 6 weeks in the absence of both BAP and TDZ. In cultures containing only 5.0 *μ*M BAP, the average number of roots was 9.1 after 4 weeks and 12.2 after 6 weeks. The presence of TDZ in the culture was completely suppressive to root formation (Figure 8).

## 4. Discussion

Liquid culture, either on shaker or held stationary, was more efficient during initiation than filter bridges or solid medium. Evaluations indicated that 2.0 *μ*M TDZ was significantly better with 5.0 *μ*M BAP intermediate than 20.0 *μ*M BAP which inhibited shoot elongation. Shoots proliferated on BAP developed roots after the second week in culture. The poor rooting ability in TDZ as compared to the previous multiplication phase indicated its repressive effect on rooting. These findings agree with earlier findings of Murashige [36]. Acheampong and Henshaw [37] observed that agitated liquid media initiated the development of protocorm-like bodies, which differentiated into plantlets upon transfer into stationary liquid media. The state of the nutrient medium apparently played a significant role in determining the pattern of organogenesis in cocoyam. Jackson et al. [38] noticed the poor growth of taro cultured on agar medium. TDZ completely suppressed root formation. Explants subcultured on Stationary Liquid Media in Erlenmeyer Flasks proliferated heavily. These findings are similar to those reported on grape by Sudarsono and Goldy [39] but contrary to that of Gray and Benton [40] who also studied grape. TDZ produced more compressed shoots, while BAP cultures produced shoots that more easily differentiated into well-defined plantlets with eventual formation of extensive root systems. In muscadine grape, rooting was impossible in the presence of BAP with higher levels affecting subsequent rooting when transferred on media without BAP [41]. Some other reports indicated that TDZ repressed root formation [40] as well. Initiation with 20 *μ*M BAP represses shoots growth, and thus proliferation. When these repressed tissues are further maintained in the same medium, their proliferation is restricted. Lee and Wetzstein [41] observed high mortality of muscadine grape shoots at 20 *μ*M BAP and higher levels. In a study to develop a rapid and efficient shoot regeneration system suitable for the transformation of lentil using TDZ, it was found that MS medium supplemented with 0.25 mg/L, TDZ produced the highest frequency of shoot formation from cotyledonary nodes in both genotypes [28]. Induction medium supplemented with 5 mgl-L BAP and 20 or 40 mg/L adenine sulphate (AS) resulted in a higher average of shoots formation when common bean was cultured using MS medium [29]. Alam and Khaleque [22] cultured groundnuts explants on MS medium with different concentration of 2,4-D, BAP, and NAA. 2,4-D at 2 mg/L was found more suitable for good callus induction. MS medium supplemented with different concentrations of BAP produced small shoot bud at different subculture and maximum number of shoot bud differentiation was observed from 2.5 mg/L BAP concentration. They concluded that 2,4-D was the best for callus induction, and BAP was found more suitable for organogenesis compared to NAA. Also, taro plants were regenerated via somatic embryogenesis and organogenesis on Murashige and Skoog (MS) medium [12] with a two-step protocol utilized combinations of 2,4-dichlorophenoxyacetic acid (2,4-D), thidiazuron (TDZ), indole-3-acetic acid (IAA), and 6-benzylaminopurine (BAP) [20]. In this study, TDZ had a tendency to enhance the initial BAP effects. The poor rooting ability in TDZ as compared to multiplication phase indicates that as long as the cultures are on TDZ, the greater its repressive effect on rooting.

The 2.0 *μ*M TDZ medium increased shoot proliferation from four to six weeks of culture. Shoot proliferation in growth regulator-free medium probably reached a maximum at four weeks. It could be better to reculture into a fresh medium at four weeks to optimize proliferation. Continued proliferation of shoots in a medium without growth regulators may be the result of the cumulative effect of growth regulators in previous media. A comparison of root formation in 2.0 *μ*M TDZ and growth regulators-free multiplication media substantiates the inhibition of rhizogenesis by TDZ, while all growth regulators-free cultures rooted. Kerns and Meyer [42] reported similar results for Acer freemanii (autumn blaze maple) cultures. Although the mode of TDZ action in organogenesis is not yet determined, it is possible that root induction is initiated in its presence, but development is delayed and expressed in its absence. Shoots were found to be proliferated in either solid or liquid media, with a tendency of getting larger numbers in solid media. These findings are in contrast to a previous report by Ng and Hahn [43] who did not find cocoyam plantlet formation in agitated liquid cultures.

All TDZ and BAP combinations resulted in an approximate doubling of shoot numbers from four to six weeks. Growth regulators levels significantly affected rooting. Virtually no roots were formed in treatments containing TDZ emphasizing its suppressive effect on rooting. Nyochembeng and Garton [4] found that thidiazuron was more favorable for callus production from petioles than shoot tips. The callus mass from petioles was significantly greater than from shoot tips. Dicamba at 1.36 *μ*M produced significantly more callus than other concentrations in the absence of TDZ, while 0.45 *μ*M stimulated mainly roots at the base of the petiole explants and later plantlets in both petioles and shoot tips. Thidiazuron had a promotive effect on callus proliferation only at higher dicamba concentrations (4.52 and 13.5 *μ*M) but delayed callus formation over the entire explants and further proliferation for six weeks compared to dicamba alone. When callus derived from different treatments was subcultured onto media containing dicamba (1.36 *μ*M), proliferation was enhanced and more than 80% became organized into shoots, roots, or a mixture of both. Callus derived from TDZ media initiated shoot organs (bud clumps and single shoot buds) first. Rapid callus initiation and multiplication in cocoyam appear to require potent auxins, that is, dicamba. In the presence of dicamba, darkness was not required for callus initiation. The potency of dicamba has been observed in other tropical monocots, for example, ginger [44] and banana [45]. Subculture of friable and rapidly growing shoot tip callus into B5 basal medium containing dicamba with or without kinetin and 2,4-D with kinetin, followed by agitation, demonstrated that: (i) cocoyam callus can form suspension cultures especially in media containing dicamba alone, and (ii) the organization of callus into bud clumps was greatly enhanced by agitation of suspension cultures of callus tissue, suggesting that the pattern of morphogenesis in cocoyam callus may also be influenced by physical factors of the medium such as aeration and medium matrix. Asokan et al. [46] observed a 3-fold increase in shoot length of *X. caracu *in liquid shaken media compared to solid media. The differentiation pattern was affected by concentration and culture method utilized. For example, the presence of auxins especially at 1.35 *μ*M (dicamba or 2,4-D) inhibited shoot formation whereas kinetin (0.46 *μ*M) stimulated shoot formation from initiated bud clumps. Several reports have mentioned the development of protocorms in shoot tip callus cultures [47–50] and directly on agitated axillary, apical, and adventitious bud cultures of cocoyam [37]. Reports on other plants indicated controversial results. Culture on elongation medium supplemented with GA3 was 55% more effective with respect to overall shoot production than that on medium without GA3 for Seedling-derived cotyledon explants of summer squash commercial cultivars True French, Ma'yan, and Goldy [16]. Various concentrations of 2,4-D and NAA were used alone or in combination. 2,4-D at 3–21 *μ*M concentrations in the culture media produced 100% callus induction from soybean-germinated seeds. Roots and shoots were obtained using BAP and Kinetin containing culture media. 5 *μ*M BAP was the most effective for that purpose [19]. In a study to develop an efficient method for shoot regeneration of canola and to compare the regeneration capacity of different explants on MS medium with several combinations of plant growth regulators, it was found that the highest shoot regeneration took place when explants were cultivated on medium, containing 1.0 mg/L NAA, 8.0 mg/L BAP and 3.0 mg/L ABA. Also, vitrification of regenerants was promoted by increasing the auxin NAA or cytokinin BAP, and ABA in the nutrient medium [25]. To determine the best combinations of plant growth regulators and other conditions in order to achieve organogenesis and multiplication directly from shoot tips of date palm without callus formation so as to avoid any possibility of undesirable genetic variability, Khierallah, and Bader [27] found that MS-modified medium supplemented with 2.0 mg/L isopentenyladenine (2ip), 1.0 mg/L benzyl adenine (BA), 1.0 mg/L naphthaleneacetic acid (NAA), and 1.0 mg/L naphthoxyacetic acid (NOA) was the best for bud formation. The maximum morphogenic callus induction rate was observed in summer squash on MS medium supplemented with 2.5 mg/L 2,4-D. The highest percentage of shoot regeneration and highest mean number of shoots per culture were obtained with 0.5 mg/L thidiazuron. Regenerated shoots were rooted in MS medium supplemented with 1.0 mg/L IBA [17]. A variety of explants of winter squash were cultured using media containing different concentrations of 6-benzylaminopurine (BA). Plant regeneration was optimal when the proximal parts of cotyledons from 4-day-old seedlings were cultured on induction medium composed of MS medium with 1 mg/L BA. Adventitious shoots were subcultured on elongation medium composed of MS medium with 0.1 mg/L BA, and the elongated shoots were successfully rooted on MS medium without growth regulators for 2 weeks [18]. Kanmegne and Omokolo [51] mentioned that organogenesis in cocoyam, when induced in the presence of a growth regulator, is preceded by an increase in soluble peroxidase activity, followed by a drop after the appearance of organs. The increase in enzyme activity can be due to the presence of the growth regulator than to organogenesis. This indicates that total peroxidase activity is not a proper marker for the orientation of morphogenesis in cocoyam. In general, during morphogenesis, cellular proteins differ in their function and in their timing and extent of expression during the process [52–54]. It has been shown in a number of plant systems that, during organogenesis, functionally related proteins seem to be encoded by groups of coordinately expressed genes and that plant growth regulators are key moderators [55–58]. Further biochemical analyses, for example, basic isoperoxidases, polyphenoloxidase, and phenol composition are needed for a better understanding of the mechanism underlying differentiation in *X. sagittifolium.*

In conclusion, cocoyam has been successfully micropropagated in vitro through various procedures: the use of either BAP, TDZ or both; agitated and stationary liquid media at the initiation stage; agitated, stationary or semisolid media at the multiplication stage; stationary liquid and semisolid media at the elongation, but increased substantially during the multiplication phases. All concentrations tested (5–20 *μ*M BAP and 1–4 *μ*M TDZ) produced significantly more axillary shoots per shoot tip than the control without cytokinins. Greater proliferation rates were obtained through the use of 20 *μ*M BAP and 2 *μ*M TDZ, respectively, 12 weeks from initiation. Shoots produced with BAP were larger and more normal in appearance than those produced with TDZ, which were small, compressed, and stunted. Based on our results, the use of stationary liquid media is recommended because of economic reasons.

## Figures and Tables

**Table 1:** Analysis of variance with mean squares and treatment significance of media substrate and growth regulator treatments effect on relative growth of cocoyam shoot tips after 4 weeks of initiation.

Source	DF	Mean squares	*P* value*
Substrate (S)	3	3.44	0.019
Growth regulators (R)	2	12.32	<0.0001
S × R	6	2.24	0.014
Rep	9	15.6	0.181

*Significant at *P* ≤ 0.05.

**Table 2:** Analysis of variance with mean squares and treatment significance of the interaction effect of TDZ and BAP on cocoyam shoot and root proliferation during multiplication after 4 and 6 weeks.

Duration	Four weeks			Six weeks		
Source	DF	Mean squares	*P* value	DF	Mean squares	*P* value*
Shoot proliferation:						
Growth regulators	5	1212.3	<0.0001	5	2212.6	<0.0001
Rep	20	2222.2	0.14	20	3256.0	0.44
Root proliferation:						
Growth regulators	5	955.5	<0.0001	5	1462.0	<0.0001
Rep	20	1120.8	0.26	20	1230.0	0.28
Shoot length:						
Growth regulators	5	3200.5	<0.0001	5	4355.0	<0.0001
Rep	20	6612.0	0.65	20	7655.0	0.57

*Significant at *P* ≤ 0.05.

## Abbreviations

MS: Murashige and Skoog (1962) medium
TDZ: Thidiazuron
BAP: Benzylaminopurine
BM: Basal medium
NAA: 1-naphthaleneacetic acid
AS: Adenine sulphate.
